# Crystal structures and Hirshfeld surface analysis of [κ^2^-*P*,*N*-{(C_6_H_5_)_2_(C_5_H_5_N)P}Re(CO)_3_Br]·2CHCl_3_ and the product of its reaction with piperidine, [*P*-{(C_6_H_5_)_2_(C_5_H_5_N)P}(C_5_H_11_N)Re(CO)_3_Br]

**DOI:** 10.1107/S2056989019008089

**Published:** 2019-06-21

**Authors:** Franco Palominos, Carolina Muñoz, Poldie Oyarzun, Marianela Saldías, Andrés Vega

**Affiliations:** a Universidad Andrés Bello, Departamento de Ciencias Químicas, Facultad de Ciencias Exactas, Quillota 980, Viña del Mar, Chile; b Laboratorio de Análisis de Sólidos, Facultad de Ciencias Exactas, Salvador Sanfuentes 2357, Santiago, Chile

**Keywords:** crystal structure, di­phenyl­pyridyl­phosphine, rhenium(I) tricarbon­yl, Hirshfeld surface analysis, two-dimensional fingerprint plots, thermogravimetric analysis

## Abstract

The coordination of the ligands with respect to the central atom in the complex bromido­tricarbon­yl[diphen­yl(pyridin-2-yl)phosphane-κ^2^ *N*,*P*]rhenium(I) chloro­form disolvate, [ReBr(C_17_H_14_NP)(CO)_3_]·2CHCl_3_ or [κ^2^-*P*,*N*-{(C_6_H_5_)_2_(C_5_H_5_N)P}Re(CO)_3_Br]·2CHCl_3_, (**I**·2CHCl_3_), is best described as a distorted octa­hedron with three carbonyls in a facial conformation, a bromide atom, and a biting *P*,*N*-di­phenyl­pyridyl­phosphine ligand. Hirshfeld surface analysis shows that C—Cl⋯H inter­actions contribute 26%, the distance of these inter­actions are between 2.895 and 3.213 Å. The reaction between **I** and piperidine (C_5_H_11_N) at 313 K in di­chloro­methane leads to the partial decoord­ination of the pyridyl­phosphine ligand, whose pyridyl group is replaced by a piperidine mol­ecule, and the complex bromido­tricarbon­yl[diphen­yl(pyridin-2-yl)phosphane-κ*P*](piperidine-κ*N*)rhenium(I), [ReBr(C_5_H_11_N)(C_17_H_14_NP)(CO)_3_] or [*P*-{(C_6_H_5_)_2_(C_5_H_5_N)P}(C_5_H_11_N)Re(CO)_3_Br] (**II**). The mol­ecule has an intra­molecular N—H⋯N hydrogen bond between the non-coordinated pyridyl nitro­gen atom and the amine hydrogen atom from piperidine with *D*⋯*A* = 2.992 (9) Å. Thermogravimetry shows that **I**·2CHCl_3_ losses 28% of its mass in a narrow range between 318 and 333 K, which is completely consistent with two solvating chloro­form mol­ecules very weakly bonded to **I**. The remaining **I** is stable at least to 573 K. In contrast, **II** seems to lose solvent and piperidine (12% of mass) between 427 and 463 K, while the additional 33% loss from this last temperature to 573 K corresponds to the release of 2-pyridyl­phosphine. The contribution to the scattering from highly disordered solvent mol­ecules in **II** was removed with the SQUEEZE routine [Spek (2015 ▸). Acta Cryst. C71, 9-18] in *PLATON*. The stated crystal data for *M*_r_, μ *etc*. do not take this solvent into account.

## Abstract 2

The reaction of the ligand di­pyridyl­phosphine with (Re(CO)_3_(OC_4_H_8_)Br)_2_ followed by crystallization in chloro­form leads to [κ^2^-*P*,*N*-{(C_6_H_5_)_2_(C_5_H_5_N)P}Re(CO)_3_Br]·2CHCl_3_. Reaction of this complex with piperidine leads to partial decoordination of the 2-pyridyl­phosphine in the product, [*P*-{(C_6_H_5_)_2_(C_5_H_5_N)P}(C_5_H_11_N)Re(CO)_3_Br], which displays an intra­molecular hydrogen bond between the piperidine aminic hydrogen atom and the uncoordinated pyridyl group, with *D*⋯*A* = 2.992 (9) Å.

## Chemical context   

Phosphine-type ligands having a second type of atom or coordinating function have been of great inter­est in many areas of chemistry. The existence of a second coordination atom with different properties, coordination capability or *trans* effect adds possibilities during a catalytic cycle (Guiry & Saunders, 2004 ▸). In particular, much attention has been paid to one of the simplest mol­ecules of this kind, di­phenyl­pyridyl­phosphine P(C_6_H_5_)_2_(C_5_H_5_N) (PPh_2_Py). The mol­ecule is a rigid bidentate ligand (Abram *et al.*, 1999 ▸; Knebel & Angelici, 1973 ▸).

The reaction of the di­phenyl­pyridyl­phosphine ligand with the rhenium dimer (Re(CO)_3_(OC_4_H_8_)Br)_2_ in chloro­form as solvent leads to the complex *P*,*N*-{(C_6_H_5_)_2_(C_5_H_5_N)P}Re(CO)_3_Br]·2CHCl_3_ (**I**·2CHCl_3_). It presents a similar structure to the widely studied [(*N*,*N*)Re(CO)_3_(*L*)] complexes, which have inter­esting photophysical and photochemical properties (Cannizzo *et al.*, 2008 ▸). Complex **I** has been shown to be a dual emitter (Pizarro *et al.*, 2015 ▸). It is also inter­esting to note that the PPh_2_Py ligand can be partially decoordinated by reaction of the complex with a monodentate ligand, like piperidine (C_5_H_11_N), leading to the complex [*P*-{(C_6_H_5_)_2_(C_5_H_5_N)P}(C_5_H_11_N)Re(CO)_3_Br] (**II**).
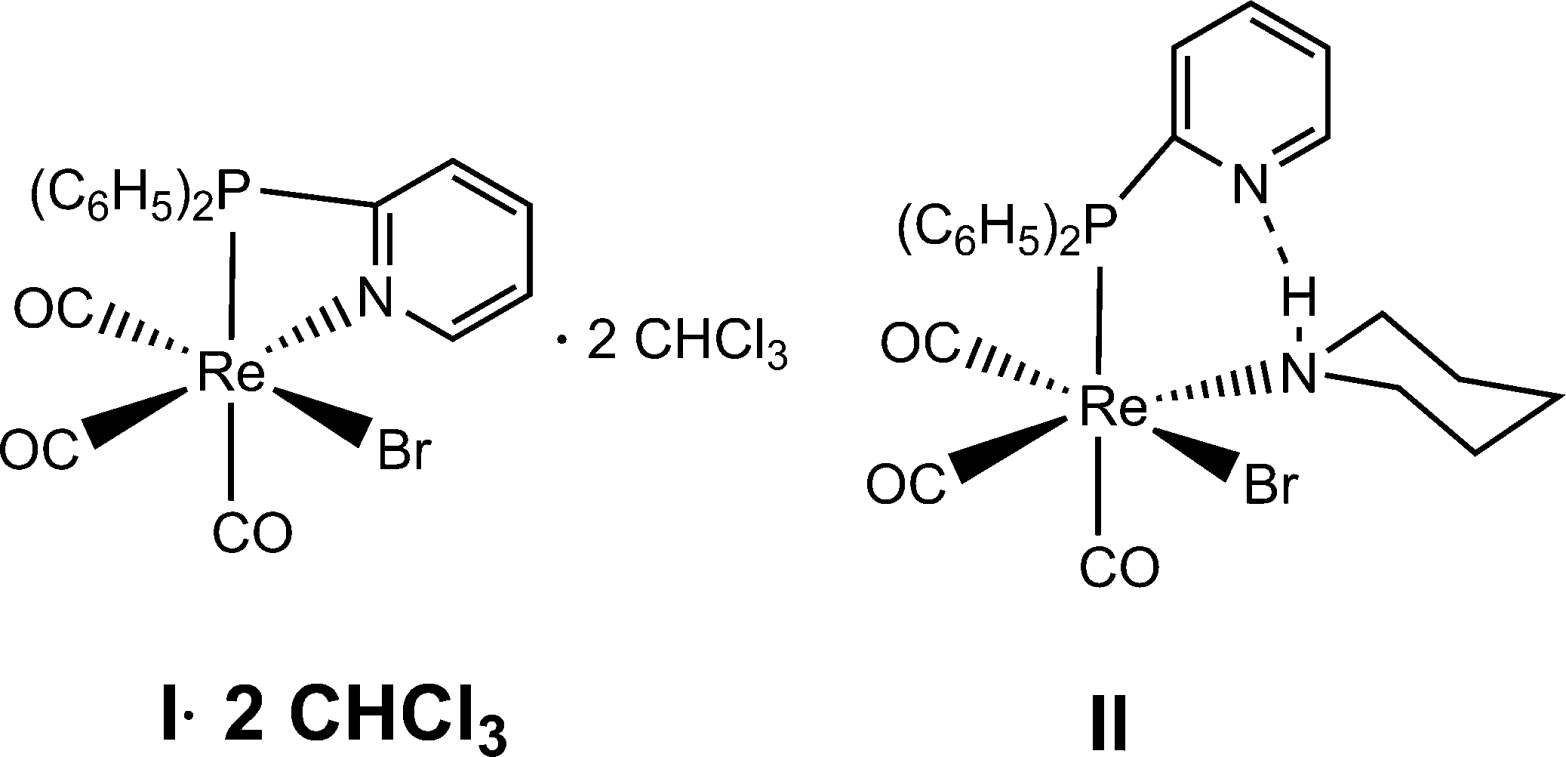


## Structural commentary   

**Figure 1:**
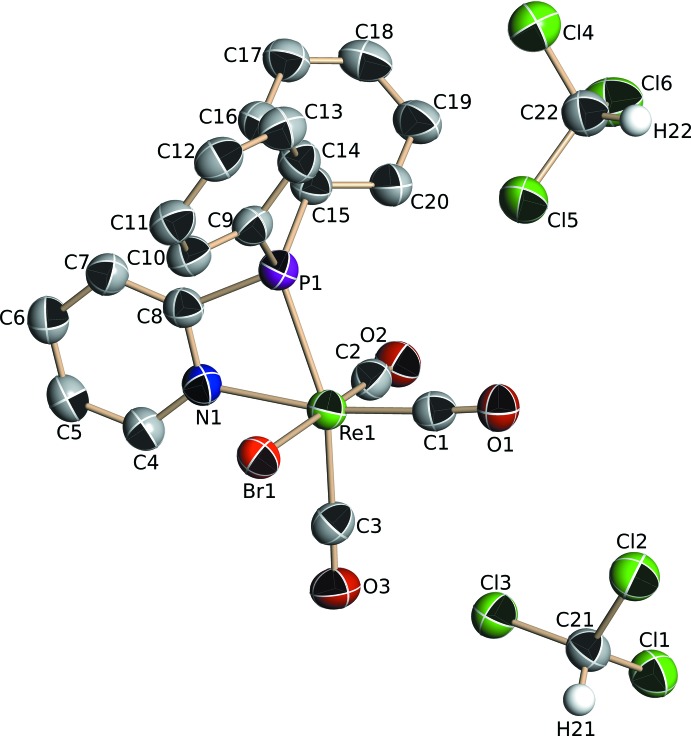
Mol­ecular view of complex **I**·2CHCl_3_, showing the numbering scheme. Displacement ellipsoids are shown at the 33% probability level. For clarity, the C-bound H atoms of **I** have been omitted.

The mononuclear Re^I^ complex **I** with a bidentate P,N (chelating) ligand crystallized from a chloro­form solution in the monoclinic space *P*2_1_/*c*. Selected geometrical data are summarized in Table 1 ▸, and the mol­ecular structure of complex **I**·2CHCl_3_ is given in Fig. 1 ▸. The coordination environment of the central rhenium atom is defined for phos­phorus and nitro­gen atoms from PPh_2_Py, a bromide atom in an apical position and three carbonyl carbon atoms in a *fac* correlation, generating a distorted octa­hedral environment. Additionally, two chloro­form mol­ecules crystallize together with the complex mol­ecule.

**Figure 2:**
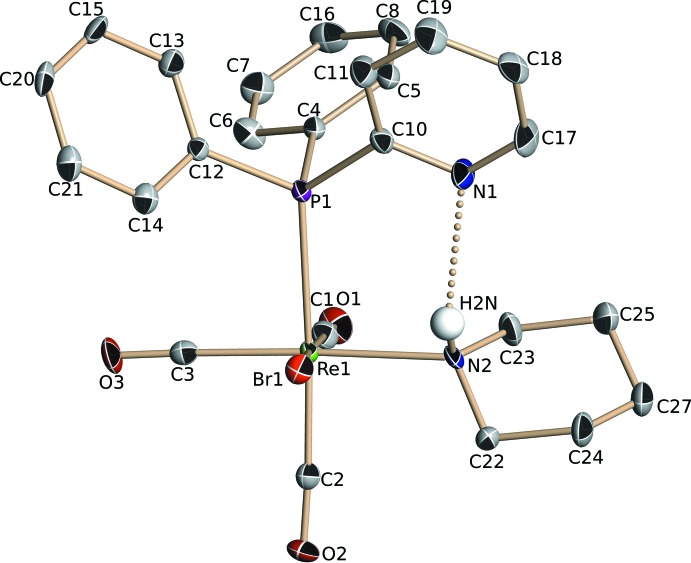
Mol­ecular view of complex **II** showing the numbering scheme. Displacement ellipsoids are shown at the 33% probability level. For clarity, the C-bound H atoms have been omitted.

The mononuclear Re^I^ complex **II**, crystallized from a CH_2_Cl_2_/CH_3_CN (2:1) solution in the triclinic space group *P* 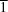. Selected geometrical data are given in Table 2 ▸, and the mol­ecular structure of the complex is illustrated in Fig. 2 ▸. The central rhenium atom displays a non-regular octa­hedral coordination geometry, with three facial carbonyl groups, a monodentate PPh_2_Py ligand, a piperidine C_5_H_11_N mol­ecule and a bromide anion. The piperidine ring displays a chair-like conformation. An intra­molecular hydrogen bond is defined between the non-coordinated pyridyl nitro­gen atom and the amine hydrogen atom from piperidine, N2—H2*N*⋯N1, with *D*⋯*A* = 2.992 (9) Å (Table 4 ▸). There are also two C—H⋯Br intra­molecular contacts present involving atom Br1 and a phenyl H atom (H14) and a methyl­ene H atom (H22*B*) of the pypridine ring (Table 4 ▸).

## Supra­molecular features   

**Figure 3:**
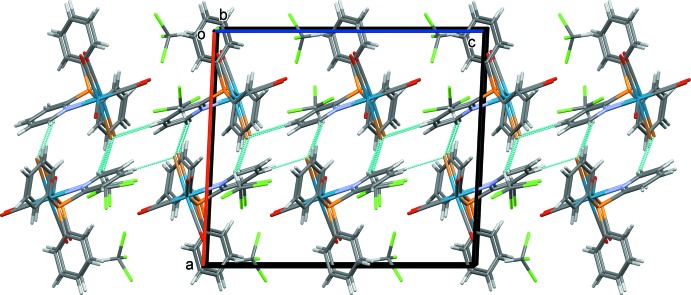
A view along the *b* axis of the crystal packing of complex **I**·2CHCl_3_. The hydrogen bonds are shown as dashed lines (see Table 3 ▸).

In the crystal of **I·2CHCl_3_,** the lattice has two solvating mol­ecules of chloro­form per complex mol­ecule. The cell has a larger volume than for the unsolvated one [2836.7 (15) *vs* 2119.2 (3) Å^3^ (Venegas *et al.*, 2011 ▸)] whose geometrical parameters are very similar to those of complex **I**. In the crystal, the chloro­form solvent mol­ecules are involved in weak C—H⋯Br hydrogen bonds and they link the complex mol­ecules to form layers lying parallel to the *bc* plane (Fig. 3 ▸ and Table 3 ▸).

**Figure 4:**
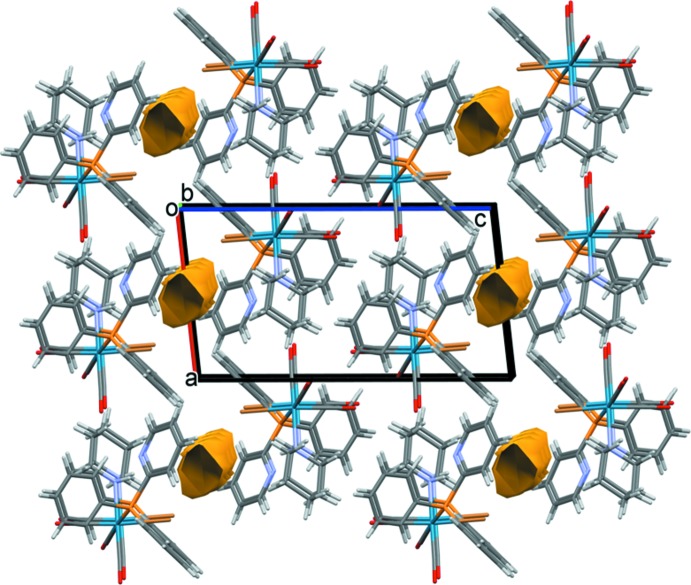
A view along the *b* axis of the crystal packing of complex **II**. The voids occupied by the disordered solvent mol­ecules are shown in yellow–brown (calculated using *Mercury*; Macrae *et al.*, 2008 ▸).

In the crystal of **II**, a region of highly disordered electron density was equated to the present of a disordered aceto­nitrile mol­ecule. The contribution to the scattering was removed with the SQUEEZE routine in *PLATON* (Spek, 2015 ▸). A view of the crystal packing, showing the regions, or voids, occupied by this disordered solvent in given in Fig. 4 ▸.

## Hirshfeld surface analysis of complex I·2CHCl_3_   

**Figure 5:**
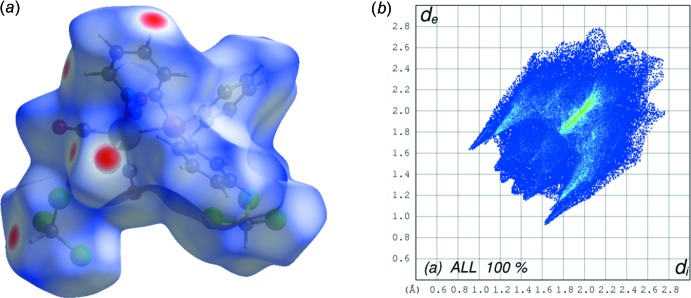
(a) The Hirshfeld surface of complex **I**·2CHCl_3_, mapped over *d*_norm_ in the range −0.2767 to +1.3337 arbitrary units. (*b*) The two-dimensional fingerprint plot of complex **I**·2CHCl_3._

In order to visualize and qu­antify the inter­molecular inter­actions in the crystal packing of complex **I**·2CHCl_3_, in particular those involving the chloro­form solvent mol­ecules, an Hirshfeld surface analysis was performed and two-dimensional fingerprint plots generated. The Hirshfeld surface analysis (Spackman & Jayatilaka, 2009 ▸) and the associated two-dimensional fingerprint plots (McKinnon *et al.*, 2007 ▸) were performed with *CrystalExplorer17* (Turner *et al.*, 2017 ▸). The Hirshfeld surface mapped over *d*_norm_ = *d*_e_ + *d*_i_, is given in Fig. 5 ▸ *a* (*d*_e_ represents the distance from the surface to the nearest nucleus external to the surface, and *d*_i_ is the distance from the surface to the nearest nucleus inter­nal to the surface). In this *d*_norm_ view (Fig. 5 ▸ *a*), blue represents the longest distances while the shortest distances are depicted as red spots (Dalal *et al.*, 2015 ▸).

**Figure 6:**
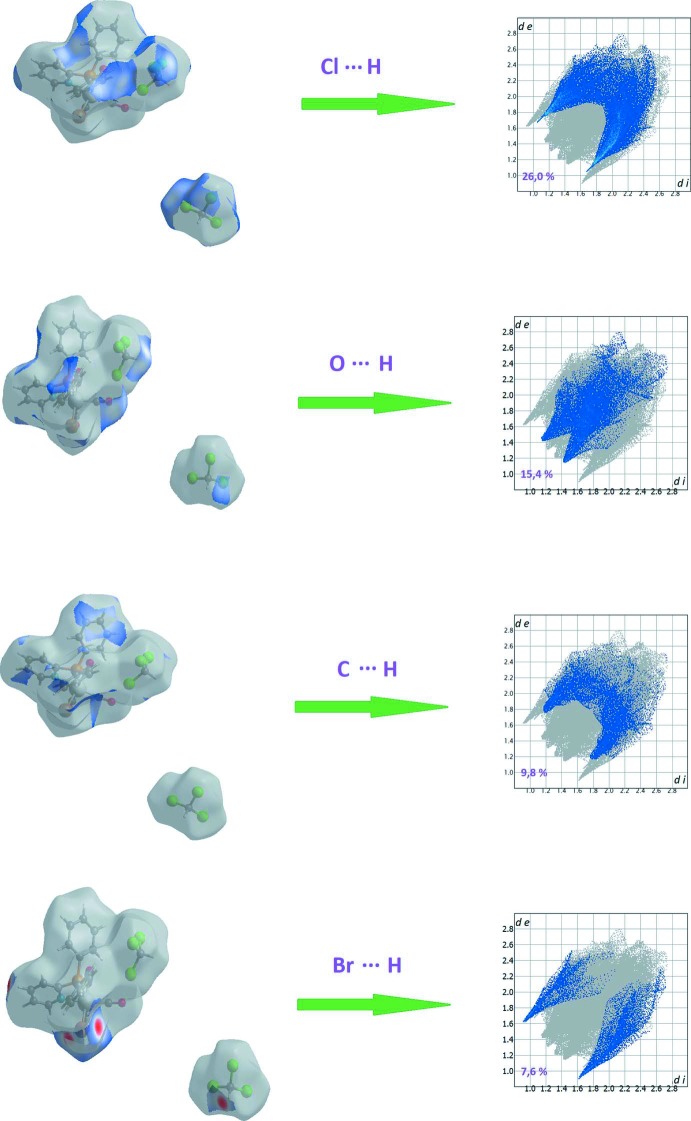
Two-dimensional fingerprint plots with a *d*_norm_ view of the Cl⋯H/H⋯Cl (26.0%), O⋯H/H⋯O (15.4%), C⋯H/H⋯C (9.8%) and Br⋯H/H⋯Br (7.6%) contacts in the coordination complex **I**·2CHCl_3_.

The two-dimensional fingerprint plot for the whole complex is given in Fig. 5 ▸ *b*. Apart from the H⋯H inter­molecular contacts that contribute *ca* 15% the other most relevant inter­molecular inter­actions, as determined from the Hirshfeld surface analysis of complex **I**·2CHCl_3_, are shown in Fig. 6 ▸. The Cl⋯H/H⋯Cl, O⋯H/H⋯O and C⋯H/H⋯C inter­actions contribute 26.0, 15.4 and 9.8%, respectively, to the Hirshfeld surface. Some distances for these inter­actions are Cl1⋯H19 = 2.90 Å, H22⋯O2 = 2.69 Å and H21⋯Br1 = 2.66 Å.

## Thermogravimetric analysis   

**Figure 7:**
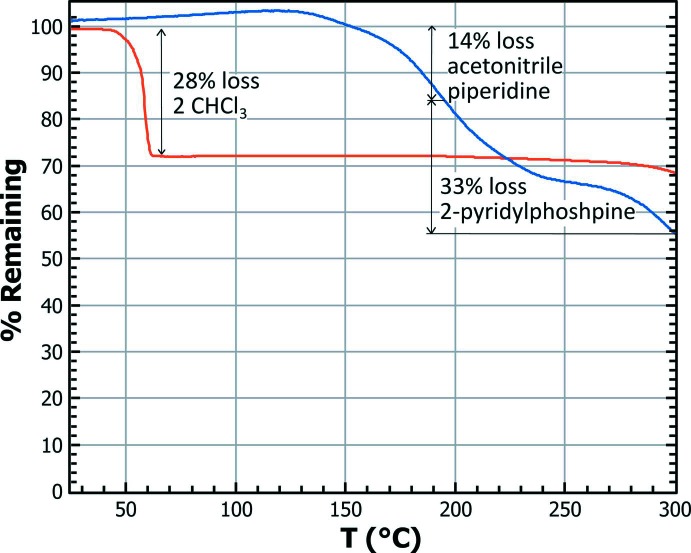
Weight loss for **I**·2CHCl_3_ (red line) and **II** (blue line) between room temperature and 300°C.

Thermogravimetric analyses from 25 to 300°C were performed for both compounds under an N_2_ flux at a heating rate of 1°C min^−1^ (see Fig. 7 ▸). Thermogravimetric analysis for compound **I**·2CHCl_3_ (Fig. 7 ▸, red line), shows that it loses 28% of its mass in a narrow range, between 45 and 60°C. This mass loss is completely consistent with the two solvating chloro­form mol­ecules detected by the crystal structure analysis. The boiling point of chloro­form, 61°C, is almost identical to the temperature where the mass loss stops, suggesting that the chloro­form mol­ecules are weakly bonded to the rhenium ones in the solid. From 60 to 300°C the remaining matrix is completely stable.

Compound **II** loses 12% of its initial mass between 154 and 190°C (Fig. 7 ▸, blue line). This loss of mass can be associated with the release of the aceto­nitrile and piperidine mol­ecules (14.7%). The relatively high temperature at which decomposition begins compared to the piperidine boiling point, 105°C, suggest that it is strongly bonded to **II**. From 190 to 300°C, another 33% of mass loss is registered, which can be associated with the release of the PPh_2_Py (36.6%, b.p.163°C).

## Database survey   

The di­phenyl­pyridyl­phosphine ligand has been extensively studied and used as a monodentate and bidentate ligand with different metals, including Ru^II^ (Ooyama & Sato, 2004 ▸) where the CO_2_-reducing properties of the complex were studied. Another Ru^II^ complex with PPh_2_Py (Kumar *et al.*, 2011 ▸) has been studied as an inhibitor of DNA-topoisomerases of the filarial parasite *S. cervi.* Re^I^–nitro­sil complexes with PPh_2_Py have been studied structurally and photophysically (Machura & Kruszynski, 2006 ▸).

Piperidine is a ligand that has been widely used with various transition metals. It has been used as a ligand with tungsten and molybdenum to study the *cis*–*trans* effect by using larger ligands and increasing the steric hindrance (Darensbourg *et al.*, 2007 ▸).

## Synthesis and crystallization   

The reagents, (Re(CO)_3_(OC_4_H_8_)Br)_2_ and (C_6_H_5_)_2_(C_5_H_5_N)P were used as provided from supplier (Aldrich), with no purification before use. Seccosolv™ solvents were used without any further purification. Standard Schlenck techniques under argon atmosphere were used for all manipulations.

**Synthesis of I**. 500 mg of (Re(CO)_3_(OC_4_H_8_)Br)_2_ (0.590 mmol) were dissolved in 5 ml of chloro­form. 312 mg of diphenyl-2-pyridyl­phosphine (1.18 mmol) was dissolved in 10 ml of chloro­form. The two solutions were mixed, changing from colourless to a translucent yellow after 10 minutes of reaction. The reaction was left to continue for a further 2 h. Addition of 2 ml of pentane to the mixture and standing by one day lead to yellow diffraction-quality crystals of **I**·2CHCl_3_ (601 mg, 82.8% yield).
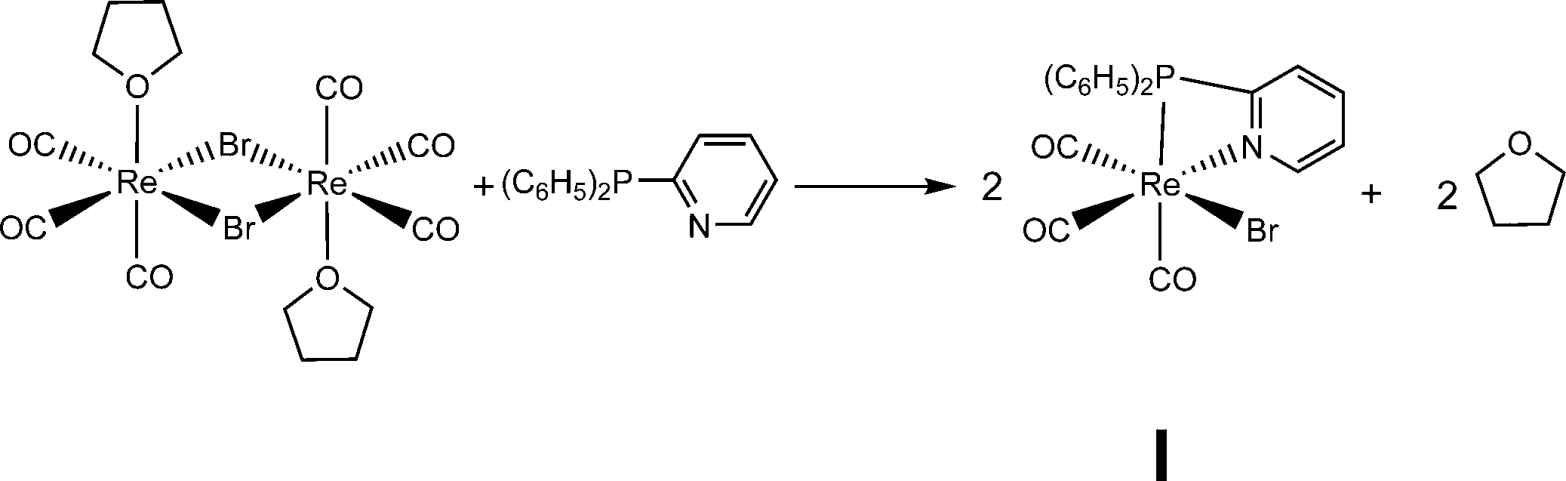


**Synthesis of II.** The compound was prepared by direct reaction between **I** and an excess of piperidine (C_5_H_11_N) at 313 K in CH_2_Cl_2_. 50.0 mg of [*P*,*N*-{(C_6_H_5_)_2_(C_5_H_5_N)P}Re(CO)_3_Br] (0.082 mmol) were dissolved in 10 ml of CH_2_Cl_2_ giving rise to a yellow solution. Then, 40 µL of piperidine (0.51 mmol) was slowly added. The reaction was allowed to continue for six days with constant agitation at 313 K. After cooling, the reaction mixture was layered with aceto­nitrile. Small orange–yellow diffraction-quality crystals were obtained after one week.
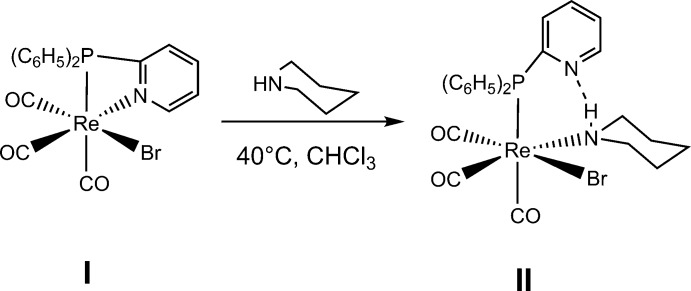


## Refinement   

Crystal data, data collection and structure refinement details are summarized in Table 5 ▸. For both compounds, the hydrogen atoms were positioned geometrically and refined using a riding model: C—H = 0.93-0.97 Å with *U*_iso_(H) = 1.2*U*_eq_(C). For **II**, the amine hydrogen atom of the piperidine ring was located in a Fourier-difference map and then subsequently refined with a distant constraint of 0.82 Å. During the last stages of the refinement of **II**, a region of highly disordered electron density was detected within the crystal structure. As no meaningful model could be achieved, SQUEEZE (Spek, 2015 ▸) was used to model the unresolved electron density resulting from the disordered solvent. 25 electrons per cell suggest, in addition to thermogravimetry, a half aceto­nitrile mol­ecule per complex mol­ecule of **II**. The contribution of this solvent was not included in the crystal data.

## Supplementary Material

Crystal structure: contains datablock(s) I-2CHCl3, II, Global. DOI: 10.1107/S2056989019008089/nk2249sup1.cif

Structure factors: contains datablock(s) I-2CHCl3. DOI: 10.1107/S2056989019008089/nk2249I-2CHCl3sup2.hkl

Structure factors: contains datablock(s) II. DOI: 10.1107/S2056989019008089/nk2249IIsup3.hkl

CCDC references: 1921165, 1921164

Additional supporting information:  crystallographic information; 3D view; checkCIF report

## Figures and Tables

**Table 1:** Selected geometric parameters (Å, °) for **I**·2CHCl_3_ [Chem scheme1]

Re1—C2	1.892 (6)	Re1—N1	2.173 (4)
Re1—C1	1.914 (5)	Re1—P1	2.4687 (13)
Re1—C3	1.943 (5)	Re1—Br1	2.6066 (8)
			
C2—Re1—C1	90.2 (2)	C3—Re1—P1	163.62 (15)
C2—Re1—C3	88.3 (2)	N1—Re1—P1	65.39 (9)
C1—Re1—C3	93.39 (19)	C2—Re1—Br1	176.20 (14)
C2—Re1—N1	93.39 (17)	C1—Re1—Br1	92.84 (14)
C1—Re1—N1	167.89 (15)	C3—Re1—Br1	89.22 (15)
C3—Re1—N1	98.27 (17)	N1—Re1—Br1	84.11 (10)
C2—Re1—P1	93.56 (13)	P1—Re1—Br1	88.01 (3)
C1—Re1—P1	102.86 (12)		

**Table 2:** Selected geometric parameters (Å, °) for **II** [Chem scheme1]

Re1—C1	1.870 (8)	Re1—N2	2.246 (6)
Re1—C2	1.918 (7)	Re1—P1	2.4915 (18)
Re1—C3	1.932 (8)	Re1—Br1	2.6430 (9)
			
C1—Re1—C2	90.1 (3)	C3—Re1—P1	89.0 (2)
C1—Re1—C3	89.4 (3)	N2—Re1—P1	93.28 (16)
C2—Re1—C3	88.0 (3)	C1—Re1—Br1	175.3 (2)
C1—Re1—N2	92.3 (3)	C2—Re1—Br1	92.1 (2)
C2—Re1—N2	89.6 (3)	C3—Re1—Br1	94.9 (2)
C3—Re1—N2	177.1 (3)	N2—Re1—Br1	83.53 (17)
C1—Re1—P1	91.6 (2)	P1—Re1—Br1	86.44 (5)
C2—Re1—P1	176.6 (2)		

**Table 3:** Hydrogen-bond geometry (Å, °) for **I**·2CHCl_3_ [Chem scheme1]

*D*—H⋯*A*	*D*—H	H⋯*A*	*D*⋯*A*	*D*—H⋯*A*
C21—H21⋯Br1^i^	0.98	2.66	3.490 (5)	143
C4—H4⋯Br1^ii^	0.93	2.80	3.552 (5)	139

Symmetry codes: (i) 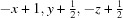; (ii) 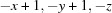.

**Table 4:** Hydrogen-bond geometry (Å, °) for **II** [Chem scheme1]

*D*—H⋯*A*	*D*—H	H⋯*A*	*D*⋯*A*	*D*—H⋯*A*
N2—H2*N*⋯N1	0.82	2.34	2.992 (9)	138
C14—H14⋯Br1	0.93	2.78	3.586 (8)	146
C22—H22*B*⋯Br1	0.97	2.83	3.499 (7)	127

**Table 5:** Experimental details

	**I**·2CHCl_3_	**II**
Crystal data		
Chemical formula	[ReBr(C_17_H_14_NP)(CO)_3_]·2CHCl_3_	[ReBr(C_5_H_11_N)(C_17_H_14_NP)(CO)_3_]
*M*_r_	852.14	698.55
Crystal system, space group	Monoclinic, *P*2_1_/*c*	Triclinic, *P* 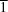
Temperature (K)	150	150
*a*, *b*, *c* (Å)	14.194 (4), 12.314 (4), 16.249 (5)	9.1384 (17), 9.8348 (18), 15.671 (3)
α, β, γ (°)	90, 92.701 (4), 90	82.956 (2), 82.047 (2), 69.765 (2)
*V* (Å^3^)	2836.7 (15)	1304.5 (4)
*Z*	4	2
Radiation type	Mo *K*α	Mo *K*α
μ (mm^−1^)	6.34	6.28
Crystal size (mm)	0.16 × 0.13 × 0.05	0.07 × 0.04 × 0.03

Data collection		
Diffractometer	Bruker SMART CCD area detector	Bruker SMART CCD area detector
Absorption correction	Multi-scan (*SADABS*; Bruker, 2012 ▸)	Numerical (*SADABS*; Bruker, 2012 ▸)
*T*_min_, *T*_max_	0.386, 0.746	0.560, 0.858
No. of measured, independent and observed [*I* > 2σ(*I*)] reflections	19979, 5550, 4526	10214, 5113, 4493
*R*_int_	0.048	0.042
(sin θ/λ)_max_ (Å^−1^)	0.617	0.617

Refinement		
*R*[*F* ^2^ > 2σ(*F* ^2^)], *wR*(*F* ^2^), *S*	0.026, 0.065, 0.99	0.042, 0.082, 1.11
No. of reflections	5550	5113
No. of parameters	317	303
No. of restraints	0	1
H-atom treatment	H-atom parameters constrained	H atoms treated by a mixture of independent and constrained refinement
Δρ_max_, Δρ_min_ (e Å^−3^)	0.39, −0.35	1.96, −1.79

Computer programs: *SMART* and *SAINT* (Bruker, 2012 ▸), *SHELXS* and *SHELXTL* (Sheldrick, 2008 ▸), *SHELXL2014* (Sheldrick, 2015 ▸), *Mercury* (Macrae *et al.*, 2008 ▸), *PLATON* (Spek, 2009 ▸) and *publCIF* (Westrip, 2010 ▸).

## supplementary crystallographic information

### Bromidotricarbonyl[diphenyl(pyridin-2-yl)phosphane-κ2N,P]rhenium(I) chloroform disolvate, [ReBr(C17H14NP)(CO)3]·2CHCl3 (I-2CHCl3) Crystal data

**Table:**

[ReBr(C_17_H_14_NP)(CO)_3_]·2CHCl_3_	*F*(000) = 1624
*M**_r_* = 852.14	*D*_x_ = 1.995 Mg m^−^^3^
Monoclinic, *P*2_1_/*c*	Mo *K*α radiation, λ = 0.71073 Å
*a* = 14.194 (4) Å	Cell parameters from 6313 reflections
*b* = 12.314 (4) Å	θ = 2.5–28.5°
*c* = 16.249 (5) Å	µ = 6.34 mm^−^^1^
β = 92.701 (4)°	*T* = 150 K
*V* = 2836.7 (15) Å^3^	Block, yellow
*Z* = 4	0.16 × 0.13 × 0.05 mm

### Bromidotricarbonyl[diphenyl(pyridin-2-yl)phosphane-κ2N,P]rhenium(I) chloroform disolvate, [ReBr(C17H14NP)(CO)3]·2CHCl3 (I-2CHCl3) Data collection

**Table:**

Bruker SMART CCD area detector diffractometer	4526 reflections with *I* > 2σ(*I*)
Radiation source: sealed tube	*R*_int_ = 0.048
phi and ω scans	θ_max_ = 26.0°, θ_min_ = 2.5°
Absorption correction: multi-scan (SADABS; Bruker, 2012)	*h* = −17→17
*T*_min_ = 0.386, *T*_max_ = 0.746	*k* = −15→15
19979 measured reflections	*l* = −19→20
5550 independent reflections	

### Bromidotricarbonyl[diphenyl(pyridin-2-yl)phosphane-κ2N,P]rhenium(I) chloroform disolvate, [ReBr(C17H14NP)(CO)3]·2CHCl3 (I-2CHCl3) Refinement

**Table:**

Refinement on *F*^2^	Secondary atom site location: difference Fourier map
Least-squares matrix: full	Hydrogen site location: inferred from neighbouring sites
*R*[*F*^2^ > 2σ(*F*^2^)] = 0.026	H-atom parameters constrained
*wR*(*F*^2^) = 0.065	*w* = 1/[σ^2^(*F*_o_^2^)] where *P* = (*F*_o_^2^ + 2*F*_c_^2^)/3
*S* = 0.99	(Δ/σ)_max_ < 0.001
5550 reflections	Δρ_max_ = 0.39 e Å^−^^3^
317 parameters	Δρ_min_ = −0.35 e Å^−^^3^
0 restraints	Extinction correction: SHELXL2014 (Sheldrick, 2015), Fc^*^=kFc[1+0.001xFc^2^λ^3^/sin(2θ)]^-1/4^
Primary atom site location: structure-invariant direct methods	Extinction coefficient: 0.00027 (8)

### Bromidotricarbonyl[diphenyl(pyridin-2-yl)phosphane-κ2N,P]rhenium(I) chloroform disolvate, [ReBr(C17H14NP)(CO)3]·2CHCl3 (I-2CHCl3) Special details

**Table:**

Geometry. All esds (except the esd in the dihedral angle between two l.s. planes) are estimated using the full covariance matrix. The cell esds are taken into account individually in the estimation of esds in distances, angles and torsion angles; correlations between esds in cell parameters are only used when they are defined by crystal symmetry. An approximate (isotropic) treatment of cell esds is used for estimating esds involving l.s. planes.

### Bromidotricarbonyl[diphenyl(pyridin-2-yl)phosphane-κ2N,P]rhenium(I) chloroform disolvate, [ReBr(C17H14NP)(CO)3]·2CHCl3 (I-2CHCl3) Fractional atomic coordinates and isotropic or equivalent isotropic displacement parameters (Å^2^)

**Table:**

	*x*	*y*	*z*	*U*_iso_*/*U*_eq_	
Re1	0.29082 (2)	0.42455 (2)	0.08046 (2)	0.06954 (7)	
Br1	0.45807 (3)	0.36073 (4)	0.13313 (3)	0.07781 (13)	
P1	0.25501 (8)	0.23375 (8)	0.04434 (7)	0.0705 (3)	
N1	0.3373 (3)	0.3742 (3)	−0.0392 (2)	0.0722 (9)	
C1	0.2470 (3)	0.4367 (3)	0.1897 (3)	0.0742 (11)	
O1	0.2190 (3)	0.4391 (3)	0.2547 (2)	0.0860 (9)	
C2	0.1733 (4)	0.4761 (3)	0.0367 (3)	0.0779 (11)	
O2	0.1045 (3)	0.5103 (3)	0.0100 (2)	0.0879 (9)	
C3	0.3368 (4)	0.5732 (4)	0.0793 (3)	0.0792 (11)	
O3	0.3635 (3)	0.6583 (3)	0.0772 (2)	0.0949 (10)	
C4	0.3826 (3)	0.4234 (4)	−0.0986 (3)	0.0783 (11)	
H4	0.3974	0.4967	−0.0932	0.094*	
C5	0.4076 (4)	0.3694 (4)	−0.1669 (3)	0.0861 (13)	
H5	0.4382	0.4061	−0.2079	0.103*	
C6	0.3879 (4)	0.2598 (4)	−0.1759 (3)	0.0855 (13)	
H6	0.4053	0.2222	−0.2224	0.103*	
C7	0.3422 (3)	0.2081 (4)	−0.1148 (3)	0.0792 (11)	
H7	0.3283	0.1344	−0.1189	0.095*	
C8	0.3171 (3)	0.2667 (3)	−0.0476 (3)	0.0701 (10)	
C9	0.3085 (3)	0.1116 (3)	0.0857 (3)	0.0735 (11)	
C10	0.4032 (4)	0.0890 (3)	0.0778 (3)	0.0788 (12)	
H10	0.4404	0.1362	0.0486	0.095*	
C11	0.4429 (4)	−0.0041 (4)	0.1134 (3)	0.0860 (13)	
H11	0.5066	−0.0187	0.1080	0.103*	
C12	0.3890 (4)	−0.0741 (4)	0.1561 (3)	0.0896 (14)	
H12	0.4161	−0.1365	0.1793	0.108*	
C13	0.2950 (4)	−0.0530 (4)	0.1651 (3)	0.0873 (14)	
H13	0.2586	−0.1006	0.1946	0.105*	
C14	0.2549 (4)	0.0392 (4)	0.1300 (3)	0.0812 (12)	
H14	0.1912	0.0532	0.1360	0.097*	
C15	0.1366 (3)	0.1951 (3)	0.0108 (3)	0.0723 (10)	
C16	0.1182 (4)	0.1207 (4)	−0.0506 (3)	0.0830 (12)	
H16	0.1680	0.0861	−0.0750	0.100*	
C17	0.0272 (4)	0.0967 (4)	−0.0765 (3)	0.0912 (14)	
H17	0.0156	0.0465	−0.1185	0.109*	
C18	−0.0466 (4)	0.1470 (4)	−0.0402 (4)	0.0987 (16)	
H18	−0.1084	0.1319	−0.0583	0.118*	
C19	−0.0293 (4)	0.2190 (4)	0.0223 (4)	0.1030 (17)	
H19	−0.0794	0.2512	0.0480	0.124*	
C20	0.0622 (4)	0.2446 (4)	0.0478 (4)	0.0910 (14)	
H20	0.0736	0.2950	0.0897	0.109*	
C21	0.3309 (4)	0.7174 (4)	0.3787 (3)	0.0853 (13)	
H21	0.3903	0.7468	0.4026	0.102*	
Cl1	0.24667 (11)	0.82254 (11)	0.37214 (10)	0.1001 (4)	
Cl2	0.29219 (12)	0.61431 (11)	0.44304 (10)	0.1061 (4)	
Cl3	0.35045 (12)	0.66739 (12)	0.28107 (9)	0.1034 (4)	
C22	0.0251 (4)	0.0957 (4)	0.3284 (4)	0.0959 (15)	
H22	0.0155	0.1018	0.3876	0.115*	
Cl4	0.02810 (13)	−0.04155 (12)	0.30240 (11)	0.1102 (4)	
Cl5	0.13277 (12)	0.15706 (16)	0.30768 (12)	0.1288 (6)	
Cl6	−0.06727 (13)	0.16078 (13)	0.27497 (13)	0.1261 (6)	

### Bromidotricarbonyl[diphenyl(pyridin-2-yl)phosphane-κ2N,P]rhenium(I) chloroform disolvate, [ReBr(C17H14NP)(CO)3]·2CHCl3 (I-2CHCl3) Atomic displacement parameters (Å^2^)

**Table:**

	*U*^11^	*U*^22^	*U*^33^	*U*^12^	*U*^13^	*U*^23^
Re1	0.06848 (12)	0.06853 (9)	0.07162 (11)	0.00019 (7)	0.00347 (7)	0.00016 (7)
Br1	0.0707 (3)	0.0804 (2)	0.0822 (3)	−0.00035 (19)	0.0024 (2)	0.0055 (2)
P1	0.0706 (7)	0.0695 (5)	0.0717 (7)	0.0009 (5)	0.0046 (5)	−0.0012 (5)
N1	0.069 (2)	0.0740 (18)	0.074 (2)	0.0018 (16)	0.0066 (17)	0.0004 (16)
C1	0.071 (3)	0.071 (2)	0.079 (3)	−0.0012 (19)	−0.005 (2)	0.000 (2)
O1	0.088 (2)	0.097 (2)	0.074 (2)	0.0047 (17)	0.0056 (17)	−0.0020 (16)
C2	0.088 (3)	0.070 (2)	0.077 (3)	0.000 (2)	0.012 (2)	−0.003 (2)
O2	0.078 (2)	0.092 (2)	0.092 (2)	0.0048 (17)	−0.0031 (18)	0.0046 (17)
C3	0.083 (3)	0.079 (2)	0.074 (3)	0.005 (2)	−0.006 (2)	−0.005 (2)
O3	0.111 (3)	0.0759 (18)	0.097 (3)	−0.0122 (18)	0.000 (2)	0.0000 (16)
C4	0.073 (3)	0.080 (2)	0.083 (3)	0.002 (2)	0.009 (2)	0.008 (2)
C5	0.076 (3)	0.099 (3)	0.083 (3)	0.002 (2)	0.013 (2)	0.010 (2)
C6	0.080 (3)	0.100 (3)	0.077 (3)	0.005 (2)	0.007 (2)	−0.002 (2)
C7	0.074 (3)	0.083 (2)	0.081 (3)	−0.001 (2)	0.002 (2)	−0.006 (2)
C8	0.064 (3)	0.071 (2)	0.074 (3)	0.0006 (17)	0.001 (2)	0.0005 (18)
C9	0.080 (3)	0.071 (2)	0.069 (3)	−0.0015 (19)	0.002 (2)	−0.0065 (18)
C10	0.080 (3)	0.071 (2)	0.085 (3)	0.001 (2)	−0.003 (2)	−0.003 (2)
C11	0.083 (3)	0.079 (2)	0.095 (4)	0.011 (2)	−0.005 (3)	−0.004 (2)
C12	0.101 (4)	0.076 (2)	0.090 (3)	0.006 (3)	−0.010 (3)	−0.002 (2)
C13	0.104 (4)	0.077 (2)	0.079 (3)	−0.003 (2)	−0.005 (3)	0.008 (2)
C14	0.081 (3)	0.081 (2)	0.082 (3)	0.003 (2)	0.003 (2)	−0.001 (2)
C15	0.072 (3)	0.0675 (19)	0.078 (3)	−0.0029 (18)	0.004 (2)	0.0041 (18)
C16	0.080 (3)	0.087 (3)	0.083 (3)	−0.010 (2)	0.006 (2)	0.000 (2)
C17	0.087 (4)	0.100 (3)	0.087 (4)	−0.015 (3)	0.001 (3)	0.000 (3)
C18	0.078 (4)	0.093 (3)	0.123 (5)	−0.014 (3)	−0.009 (3)	0.010 (3)
C19	0.073 (4)	0.090 (3)	0.146 (5)	−0.003 (3)	0.014 (3)	−0.006 (3)
C20	0.079 (3)	0.081 (3)	0.115 (4)	−0.003 (2)	0.018 (3)	−0.013 (3)
C21	0.085 (3)	0.088 (3)	0.084 (3)	−0.008 (2)	0.007 (3)	0.006 (2)
Cl1	0.1005 (10)	0.0898 (7)	0.1106 (10)	0.0026 (6)	0.0119 (8)	0.0115 (7)
Cl2	0.1271 (13)	0.0897 (7)	0.1023 (10)	−0.0028 (7)	0.0146 (9)	0.0158 (7)
Cl3	0.1147 (11)	0.1028 (8)	0.0938 (9)	−0.0140 (8)	0.0153 (8)	−0.0114 (7)
C22	0.087 (4)	0.108 (3)	0.092 (4)	−0.008 (3)	−0.001 (3)	−0.006 (3)
Cl4	0.1192 (12)	0.1039 (8)	0.1067 (11)	0.0020 (8)	−0.0035 (9)	0.0022 (8)
Cl5	0.1014 (12)	0.1516 (14)	0.1350 (14)	−0.0353 (10)	0.0217 (10)	−0.0418 (11)
Cl6	0.1167 (13)	0.1064 (9)	0.1519 (16)	−0.0004 (9)	−0.0286 (11)	0.0013 (9)

### Bromidotricarbonyl[diphenyl(pyridin-2-yl)phosphane-κ2N,P]rhenium(I) chloroform disolvate, [ReBr(C17H14NP)(CO)3]·2CHCl3 (I-2CHCl3) Geometric parameters (Å, º)

**Table:**

Re1—C2	1.892 (6)	C11—C12	1.364 (7)
Re1—C1	1.914 (5)	C11—H11	0.9300
Re1—C3	1.943 (5)	C12—C13	1.373 (8)
Re1—N1	2.173 (4)	C12—H12	0.9300
Re1—P1	2.4687 (13)	C13—C14	1.382 (6)
Re1—Br1	2.6066 (8)	C13—H13	0.9300
P1—C9	1.800 (5)	C14—H14	0.9300
P1—C15	1.806 (5)	C15—C16	1.370 (6)
P1—C8	1.815 (5)	C15—C20	1.382 (6)
N1—C4	1.330 (5)	C16—C17	1.371 (7)
N1—C8	1.360 (5)	C16—H16	0.9300
C1—O1	1.147 (5)	C17—C18	1.374 (8)
C2—O2	1.131 (6)	C17—H17	0.9300
C3—O3	1.116 (5)	C18—C19	1.362 (8)
C4—C5	1.355 (7)	C18—H18	0.9300
C4—H4	0.9300	C19—C20	1.381 (8)
C5—C6	1.384 (7)	C19—H19	0.9300
C5—H5	0.9300	C20—H20	0.9300
C6—C7	1.368 (7)	C21—Cl3	1.737 (5)
C6—H6	0.9300	C21—Cl2	1.749 (5)
C7—C8	1.370 (6)	C21—Cl1	1.762 (5)
C7—H7	0.9300	C21—H21	0.9800
C9—C10	1.385 (6)	C22—Cl6	1.735 (6)
C9—C14	1.394 (6)	C22—Cl4	1.743 (6)
C10—C11	1.392 (6)	C22—Cl5	1.751 (6)
C10—H10	0.9300	C22—H22	0.9800
			
C2—Re1—C1	90.2 (2)	C9—C10—C11	120.2 (5)
C2—Re1—C3	88.3 (2)	C9—C10—H10	119.9
C1—Re1—C3	93.39 (19)	C11—C10—H10	119.9
C2—Re1—N1	93.39 (17)	C12—C11—C10	120.5 (5)
C1—Re1—N1	167.89 (15)	C12—C11—H11	119.8
C3—Re1—N1	98.27 (17)	C10—C11—H11	119.8
C2—Re1—P1	93.56 (13)	C11—C12—C13	120.3 (5)
C1—Re1—P1	102.86 (12)	C11—C12—H12	119.9
C3—Re1—P1	163.62 (15)	C13—C12—H12	119.9
N1—Re1—P1	65.39 (9)	C12—C13—C14	119.8 (5)
C2—Re1—Br1	176.20 (14)	C12—C13—H13	120.1
C1—Re1—Br1	92.84 (14)	C14—C13—H13	120.1
C3—Re1—Br1	89.22 (15)	C13—C14—C9	120.9 (5)
N1—Re1—Br1	84.11 (10)	C13—C14—H14	119.5
P1—Re1—Br1	88.01 (3)	C9—C14—H14	119.5
C9—P1—C15	105.2 (2)	C16—C15—C20	119.2 (5)
C9—P1—C8	106.3 (2)	C16—C15—P1	122.6 (4)
C15—P1—C8	106.9 (2)	C20—C15—P1	118.2 (4)
C9—P1—Re1	128.88 (16)	C15—C16—C17	120.8 (5)
C15—P1—Re1	120.04 (14)	C15—C16—H16	119.6
C8—P1—Re1	83.14 (13)	C17—C16—H16	119.6
C4—N1—C8	118.5 (4)	C16—C17—C18	119.8 (5)
C4—N1—Re1	134.2 (3)	C16—C17—H17	120.1
C8—N1—Re1	107.2 (3)	C18—C17—H17	120.1
O1—C1—Re1	176.7 (4)	C19—C18—C17	119.9 (5)
O2—C2—Re1	177.7 (4)	C19—C18—H18	120.1
O3—C3—Re1	178.8 (5)	C17—C18—H18	120.1
N1—C4—C5	121.7 (4)	C18—C19—C20	120.5 (5)
N1—C4—H4	119.2	C18—C19—H19	119.8
C5—C4—H4	119.2	C20—C19—H19	119.8
C4—C5—C6	120.3 (5)	C19—C20—C15	119.7 (5)
C4—C5—H5	119.8	C19—C20—H20	120.1
C6—C5—H5	119.8	C15—C20—H20	120.1
C7—C6—C5	118.6 (5)	Cl3—C21—Cl2	110.9 (3)
C7—C6—H6	120.7	Cl3—C21—Cl1	110.0 (3)
C5—C6—H6	120.7	Cl2—C21—Cl1	109.8 (3)
C6—C7—C8	118.9 (4)	Cl3—C21—H21	108.7
C6—C7—H7	120.6	Cl2—C21—H21	108.7
C8—C7—H7	120.6	Cl1—C21—H21	108.7
N1—C8—C7	122.0 (4)	Cl6—C22—Cl4	110.7 (3)
N1—C8—P1	104.1 (3)	Cl6—C22—Cl5	110.5 (3)
C7—C8—P1	133.9 (3)	Cl4—C22—Cl5	109.9 (3)
C10—C9—C14	118.4 (4)	Cl6—C22—H22	108.6
C10—C9—P1	121.8 (3)	Cl4—C22—H22	108.6
C14—C9—P1	119.7 (4)	Cl5—C22—H22	108.6
			
C8—N1—C4—C5	0.8 (7)	C14—C9—C10—C11	0.1 (7)
Re1—N1—C4—C5	176.3 (4)	P1—C9—C10—C11	177.4 (4)
N1—C4—C5—C6	−1.2 (8)	C9—C10—C11—C12	0.2 (8)
C4—C5—C6—C7	0.5 (8)	C10—C11—C12—C13	−0.5 (8)
C5—C6—C7—C8	0.4 (7)	C11—C12—C13—C14	0.5 (8)
C4—N1—C8—C7	0.2 (7)	C12—C13—C14—C9	−0.2 (8)
Re1—N1—C8—C7	−176.4 (4)	C10—C9—C14—C13	−0.1 (7)
C4—N1—C8—P1	−179.2 (3)	P1—C9—C14—C13	−177.5 (4)
Re1—N1—C8—P1	4.2 (3)	C9—P1—C15—C16	−63.3 (4)
C6—C7—C8—N1	−0.8 (7)	C8—P1—C15—C16	49.5 (4)
C6—C7—C8—P1	178.3 (4)	Re1—P1—C15—C16	141.4 (3)
C9—P1—C8—N1	−132.1 (3)	C9—P1—C15—C20	118.0 (4)
C15—P1—C8—N1	115.8 (3)	C8—P1—C15—C20	−129.2 (4)
Re1—P1—C8—N1	−3.5 (3)	Re1—P1—C15—C20	−37.3 (4)
C9—P1—C8—C7	48.6 (5)	C20—C15—C16—C17	1.3 (7)
C15—P1—C8—C7	−63.4 (5)	P1—C15—C16—C17	−177.4 (4)
Re1—P1—C8—C7	177.2 (5)	C15—C16—C17—C18	−0.5 (8)
C15—P1—C9—C10	145.3 (4)	C16—C17—C18—C19	−1.2 (9)
C8—P1—C9—C10	32.1 (4)	C17—C18—C19—C20	2.1 (9)
Re1—P1—C9—C10	−62.3 (4)	C18—C19—C20—C15	−1.3 (9)
C15—P1—C9—C14	−37.4 (4)	C16—C15—C20—C19	−0.3 (8)
C8—P1—C9—C14	−150.6 (4)	P1—C15—C20—C19	178.4 (4)
Re1—P1—C9—C14	115.0 (4)		

### Bromidotricarbonyl[diphenyl(pyridin-2-yl)phosphane-κ2N,P]rhenium(I) chloroform disolvate, [ReBr(C17H14NP)(CO)3]·2CHCl3 (I-2CHCl3) Hydrogen-bond geometry (Å, º)

**Table:**

*D*—H···*A*	*D*—H	H···*A*	*D*···*A*	*D*—H···*A*
C21—H21···Br1^i^	0.98	2.66	3.490 (5)	143
C4—H4···Br1^ii^	0.93	2.80	3.552 (5)	139

Symmetry codes: (i) −*x*+1, *y*+1/2, −*z*+1/2; (ii) −*x*+1, −*y*+1, −*z*.

### Bromidotricarbonyl[diphenyl(pyridin-2-yl)phosphane-κP](piperidine-κN)rhenium(I) (II) Crystal data

**Table:**

[ReBr(C_5_H_11_N)(C_17_H_14_NP)(CO)_3_]·[+solvent]	*Z* = 2
*M**_r_* = 698.55	*F*(000) = 676.0
Triclinic, *P*1	*D*_x_ = 1.778 Mg m^−^^3^
*a* = 9.1384 (17) Å	Mo *K*α radiation, λ = 0.71073 Å
*b* = 9.8348 (18) Å	Cell parameters from 4721 reflections
*c* = 15.671 (3) Å	θ = 2.4–22.8°
α = 82.956 (2)°	µ = 6.28 mm^−^^1^
β = 82.047 (2)°	*T* = 150 K
γ = 69.765 (2)°	Stick, orange
*V* = 1304.5 (4) Å^3^	0.07 × 0.04 × 0.03 mm

### Bromidotricarbonyl[diphenyl(pyridin-2-yl)phosphane-κP](piperidine-κN)rhenium(I) (II) Data collection

**Table:**

Bruker SMART CCD area detector diffractometer	4493 reflections with *I* > 2σ(*I*)
Radiation source: sealed tube	*R*_int_ = 0.042
phi and ω scans	θ_max_ = 26.0°, θ_min_ = 2.2°
Absorption correction: numerical (SADABS; Bruker, 2012)	*h* = −11→11
*T*_min_ = 0.560, *T*_max_ = 0.858	*k* = −12→12
10214 measured reflections	*l* = −19→19
5113 independent reflections	

### Bromidotricarbonyl[diphenyl(pyridin-2-yl)phosphane-κP](piperidine-κN)rhenium(I) (II) Refinement

**Table:**

Refinement on *F*^2^	Secondary atom site location: difference Fourier map
Least-squares matrix: full	Hydrogen site location: inferred from neighbouring sites
*R*[*F*^2^ > 2σ(*F*^2^)] = 0.042	H atoms treated by a mixture of independent and constrained refinement
*wR*(*F*^2^) = 0.082	*w* = 1/[σ^2^(*F*_o_^2^) + 9.4699*P*] where *P* = (*F*_o_^2^ + 2*F*_c_^2^)/3
*S* = 1.11	(Δ/σ)_max_ = 0.001
5113 reflections	Δρ_max_ = 1.96 e Å^−^^3^
303 parameters	Δρ_min_ = −1.79 e Å^−^^3^
1 restraint	Extinction correction: SHELXL2014 (Sheldrick, 2015), Fc^*^=kFc[1+0.001xFc^2^λ^3^/sin(2θ)]^-1/4^
Primary atom site location: structure-invariant direct methods	Extinction coefficient: 0.00125 (18)

### Bromidotricarbonyl[diphenyl(pyridin-2-yl)phosphane-κP](piperidine-κN)rhenium(I) (II) Special details

**Table:**

Geometry. All esds (except the esd in the dihedral angle between two l.s. planes) are estimated using the full covariance matrix. The cell esds are taken into account individually in the estimation of esds in distances, angles and torsion angles; correlations between esds in cell parameters are only used when they are defined by crystal symmetry. An approximate (isotropic) treatment of cell esds is used for estimating esds involving l.s. planes.

### Bromidotricarbonyl[diphenyl(pyridin-2-yl)phosphane-κP](piperidine-κN)rhenium(I) (II) Fractional atomic coordinates and isotropic or equivalent isotropic displacement parameters (Å^2^)

**Table:**

	*x*	*y*	*z*	*U*_iso_*/*U*_eq_	
Re1	0.83162 (3)	0.70525 (3)	0.70289 (2)	0.01774 (10)	
Br1	0.80946 (9)	0.74617 (8)	0.86842 (5)	0.03004 (19)	
P1	0.7341 (2)	0.49823 (19)	0.75160 (12)	0.0202 (4)	
O1	0.8277 (7)	0.6670 (7)	0.5117 (4)	0.0422 (15)	
O3	1.1681 (6)	0.4948 (6)	0.6944 (4)	0.0390 (14)	
C1	0.8293 (9)	0.6804 (8)	0.5867 (5)	0.0311 (18)	
C3	1.0438 (9)	0.5701 (8)	0.6993 (5)	0.0262 (17)	
O2	0.9776 (6)	0.9453 (6)	0.6454 (4)	0.0403 (14)	
C2	0.9177 (8)	0.8584 (8)	0.6688 (5)	0.0272 (17)	
N1	0.4375 (7)	0.6604 (7)	0.8008 (5)	0.0404 (18)	
C10	0.5477 (8)	0.5329 (8)	0.8209 (5)	0.0258 (16)	
C12	0.8652 (8)	0.3427 (7)	0.8099 (4)	0.0212 (15)	
C4	0.7011 (8)	0.4161 (7)	0.6619 (5)	0.0226 (15)	
C13	0.8598 (9)	0.2025 (8)	0.8069 (5)	0.0294 (17)	
H13	0.7924	0.1883	0.7722	0.035*	
C5	0.5513 (8)	0.4262 (8)	0.6451 (5)	0.0271 (17)	
H5	0.4635	0.4741	0.6812	0.032*	
C6	0.8295 (9)	0.3428 (9)	0.6070 (5)	0.0329 (19)	
H6	0.9298	0.3356	0.6175	0.039*	
C14	0.9678 (8)	0.3586 (8)	0.8629 (5)	0.0288 (17)	
H14	0.9738	0.4502	0.8667	0.035*	
C15	0.9527 (9)	0.0861 (8)	0.8545 (5)	0.035 (2)	
H15	0.9469	−0.0058	0.8516	0.042*	
C11	0.5179 (9)	0.4359 (9)	0.8863 (5)	0.037 (2)	
H11	0.5958	0.3487	0.9002	0.044*	
C7	0.8101 (10)	0.2809 (9)	0.5372 (5)	0.038 (2)	
H7	0.8971	0.2322	0.5009	0.045*	
C8	0.5355 (10)	0.3635 (9)	0.5731 (5)	0.038 (2)	
H8	0.4361	0.3716	0.5607	0.046*	
N2	0.5893 (6)	0.8705 (7)	0.7094 (4)	0.0267 (14)	
C20	1.0531 (8)	0.1025 (8)	0.9058 (5)	0.0312 (19)	
H20	1.1158	0.0226	0.9375	0.037*	
C22	0.5748 (8)	1.0155 (7)	0.7304 (5)	0.0265 (17)	
H22A	0.6209	1.0630	0.6815	0.032*	
H22B	0.6333	1.0066	0.7791	0.032*	
C21	1.0610 (9)	0.2390 (9)	0.9101 (5)	0.0330 (19)	
H21	1.1293	0.2509	0.9451	0.040*	
C16	0.6639 (11)	0.2905 (10)	0.5211 (6)	0.042 (2)	
H16	0.6518	0.2470	0.4743	0.050*	
C18	0.2572 (9)	0.5974 (9)	0.9089 (5)	0.038 (2)	
H18	0.1558	0.6203	0.9366	0.046*	
C17	0.2958 (9)	0.6904 (10)	0.8455 (7)	0.052 (3)	
H17	0.2198	0.7794	0.8325	0.063*	
C23	0.4992 (9)	0.8828 (9)	0.6357 (6)	0.038 (2)	
H23A	0.5059	0.7862	0.6237	0.045*	
H23B	0.5463	0.9251	0.5848	0.045*	
C19	0.3699 (10)	0.4708 (10)	0.9308 (6)	0.046 (2)	
H19	0.3477	0.4074	0.9756	0.056*	
C24	0.4037 (9)	1.1105 (9)	0.7526 (6)	0.037 (2)	
H24A	0.3600	1.0682	0.8047	0.045*	
H24B	0.3999	1.2067	0.7637	0.045*	
C25	0.3279 (9)	0.9750 (9)	0.6520 (6)	0.043 (2)	
H25A	0.2777	0.9872	0.5996	0.052*	
H25B	0.2763	0.9245	0.6966	0.052*	
C27	0.3068 (9)	1.1232 (9)	0.6799 (6)	0.042 (2)	
H3A	0.3377	1.1818	0.6312	0.051*	
H3B	0.1971	1.1717	0.6986	0.051*	
H2N	0.567 (8)	0.828 (5)	0.755 (2)	0.08 (4)*	

### Bromidotricarbonyl[diphenyl(pyridin-2-yl)phosphane-κP](piperidine-κN)rhenium(I) (II) Atomic displacement parameters (Å^2^)

**Table:**

	*U*^11^	*U*^22^	*U*^33^	*U*^12^	*U*^13^	*U*^23^
Re1	0.01153 (15)	0.01837 (15)	0.02204 (17)	−0.00440 (10)	−0.00090 (10)	0.00044 (10)
Br1	0.0329 (4)	0.0300 (4)	0.0260 (4)	−0.0077 (3)	−0.0048 (3)	−0.0040 (3)
P1	0.0141 (9)	0.0199 (9)	0.0252 (10)	−0.0052 (7)	−0.0009 (7)	0.0008 (8)
O1	0.042 (4)	0.057 (4)	0.028 (3)	−0.018 (3)	−0.009 (3)	0.004 (3)
O3	0.013 (3)	0.045 (3)	0.051 (4)	−0.001 (3)	0.000 (2)	−0.005 (3)
C1	0.023 (4)	0.034 (4)	0.033 (5)	−0.009 (3)	0.003 (3)	0.001 (4)
C3	0.031 (4)	0.025 (4)	0.025 (4)	−0.012 (4)	−0.003 (3)	−0.002 (3)
O2	0.031 (3)	0.035 (3)	0.061 (4)	−0.022 (3)	0.001 (3)	−0.001 (3)
C2	0.023 (4)	0.024 (4)	0.032 (4)	−0.005 (3)	0.000 (3)	−0.004 (3)
N1	0.018 (3)	0.031 (4)	0.060 (5)	−0.002 (3)	0.013 (3)	0.009 (3)
C10	0.018 (4)	0.028 (4)	0.030 (4)	−0.008 (3)	0.002 (3)	−0.006 (3)
C12	0.017 (3)	0.021 (4)	0.023 (4)	−0.005 (3)	0.002 (3)	0.001 (3)
C4	0.019 (4)	0.021 (4)	0.028 (4)	−0.006 (3)	−0.005 (3)	0.002 (3)
C13	0.027 (4)	0.026 (4)	0.032 (4)	−0.006 (3)	−0.002 (3)	−0.001 (3)
C5	0.023 (4)	0.025 (4)	0.032 (4)	−0.008 (3)	−0.006 (3)	0.003 (3)
C6	0.032 (4)	0.047 (5)	0.023 (4)	−0.016 (4)	0.003 (3)	−0.011 (4)
C14	0.026 (4)	0.031 (4)	0.025 (4)	−0.005 (3)	0.001 (3)	−0.005 (3)
C15	0.030 (4)	0.014 (4)	0.050 (5)	0.001 (3)	0.002 (4)	0.004 (3)
C11	0.026 (4)	0.038 (5)	0.040 (5)	−0.007 (4)	0.006 (4)	0.002 (4)
C7	0.042 (5)	0.039 (5)	0.032 (5)	−0.012 (4)	0.002 (4)	−0.013 (4)
C8	0.037 (5)	0.048 (5)	0.039 (5)	−0.025 (4)	−0.018 (4)	0.010 (4)
N2	0.011 (3)	0.028 (3)	0.039 (4)	−0.008 (3)	−0.002 (3)	0.007 (3)
C20	0.021 (4)	0.028 (4)	0.029 (4)	0.006 (3)	0.002 (3)	0.005 (3)
C22	0.024 (4)	0.020 (4)	0.036 (4)	−0.007 (3)	−0.006 (3)	−0.002 (3)
C21	0.027 (4)	0.040 (5)	0.026 (4)	−0.002 (4)	−0.005 (3)	−0.001 (4)
C16	0.058 (6)	0.048 (5)	0.031 (5)	−0.029 (5)	−0.021 (4)	0.000 (4)
C18	0.024 (4)	0.046 (5)	0.043 (5)	−0.014 (4)	0.014 (4)	−0.012 (4)
C17	0.021 (4)	0.039 (5)	0.076 (7)	0.005 (4)	0.019 (4)	−0.001 (5)
C23	0.022 (4)	0.040 (5)	0.051 (6)	−0.004 (4)	−0.014 (4)	−0.010 (4)
C19	0.033 (5)	0.055 (6)	0.042 (5)	−0.014 (4)	0.013 (4)	0.006 (4)
C24	0.022 (4)	0.032 (4)	0.048 (5)	0.003 (3)	−0.001 (4)	−0.003 (4)
C25	0.032 (5)	0.035 (5)	0.063 (6)	−0.007 (4)	−0.021 (4)	0.004 (4)
C27	0.023 (4)	0.034 (5)	0.061 (6)	0.000 (4)	−0.008 (4)	0.003 (4)

### Bromidotricarbonyl[diphenyl(pyridin-2-yl)phosphane-κP](piperidine-κN)rhenium(I) (II) Geometric parameters (Å, º)

**Table:**

Re1—C1	1.870 (8)	C7—C16	1.363 (11)
Re1—C2	1.918 (7)	C7—H7	0.9300
Re1—C3	1.932 (8)	C8—C16	1.361 (12)
Re1—N2	2.246 (6)	C8—H8	0.9300
Re1—P1	2.4915 (18)	N2—C22	1.460 (9)
Re1—Br1	2.6430 (9)	N2—C23	1.479 (10)
P1—C4	1.812 (7)	N2—H2N	0.8200 (10)
P1—C12	1.817 (7)	C20—C21	1.379 (11)
P1—C10	1.837 (7)	C20—H20	0.9300
O1—C1	1.202 (9)	C22—C24	1.536 (10)
O3—C3	1.120 (8)	C22—H22A	0.9700
O2—C2	1.164 (8)	C22—H22B	0.9700
N1—C17	1.338 (9)	C21—H21	0.9300
N1—C10	1.343 (9)	C16—H16	0.9300
C10—C11	1.377 (10)	C18—C19	1.354 (11)
C12—C14	1.394 (10)	C18—C17	1.361 (12)
C12—C13	1.404 (10)	C18—H18	0.9300
C4—C6	1.387 (10)	C17—H17	0.9300
C4—C5	1.398 (9)	C23—C25	1.517 (10)
C13—C15	1.371 (10)	C23—H23A	0.9700
C13—H13	0.9300	C23—H23B	0.9700
C5—C8	1.396 (11)	C19—H19	0.9300
C5—H5	0.9300	C24—C27	1.505 (12)
C6—C7	1.375 (10)	C24—H24A	0.9700
C6—H6	0.9300	C24—H24B	0.9700
C14—C21	1.387 (10)	C25—C27	1.514 (11)
C14—H14	0.9300	C25—H25A	0.9700
C15—C20	1.362 (11)	C25—H25B	0.9700
C15—H15	0.9300	C27—H3A	0.9700
C11—C19	1.382 (10)	C27—H3B	0.9700
C11—H11	0.9300		
			
C1—Re1—C2	90.1 (3)	C16—C8—H8	119.6
C1—Re1—C3	89.4 (3)	C5—C8—H8	119.6
C2—Re1—C3	88.0 (3)	C22—N2—C23	109.6 (6)
C1—Re1—N2	92.3 (3)	C22—N2—Re1	116.4 (4)
C2—Re1—N2	89.6 (3)	C23—N2—Re1	116.1 (5)
C3—Re1—N2	177.1 (3)	C22—N2—H2N	106 (4)
C1—Re1—P1	91.6 (2)	C23—N2—H2N	118 (5)
C2—Re1—P1	176.6 (2)	Re1—N2—H2N	89 (5)
C3—Re1—P1	89.0 (2)	C15—C20—C21	119.3 (7)
N2—Re1—P1	93.28 (16)	C15—C20—H20	120.4
C1—Re1—Br1	175.3 (2)	C21—C20—H20	120.4
C2—Re1—Br1	92.1 (2)	N2—C22—C24	112.6 (6)
C3—Re1—Br1	94.9 (2)	N2—C22—H22A	109.1
N2—Re1—Br1	83.53 (17)	C24—C22—H22A	109.1
P1—Re1—Br1	86.44 (5)	N2—C22—H22B	109.1
C4—P1—C12	102.0 (3)	C24—C22—H22B	109.1
C4—P1—C10	102.6 (3)	H22A—C22—H22B	107.8
C12—P1—C10	102.6 (3)	C20—C21—C14	120.6 (8)
C4—P1—Re1	112.4 (2)	C20—C21—H21	119.7
C12—P1—Re1	116.4 (2)	C14—C21—H21	119.7
C10—P1—Re1	118.7 (2)	C8—C16—C7	120.3 (8)
O1—C1—Re1	178.8 (7)	C8—C16—H16	119.8
O3—C3—Re1	177.3 (7)	C7—C16—H16	119.8
O2—C2—Re1	175.9 (6)	C19—C18—C17	118.4 (7)
C17—N1—C10	118.0 (7)	C19—C18—H18	120.8
N1—C10—C11	121.6 (7)	C17—C18—H18	120.8
N1—C10—P1	114.4 (5)	N1—C17—C18	123.4 (8)
C11—C10—P1	124.0 (6)	N1—C17—H17	118.3
C14—C12—C13	117.5 (7)	C18—C17—H17	118.3
C14—C12—P1	121.7 (5)	N2—C23—C25	113.0 (7)
C13—C12—P1	120.7 (5)	N2—C23—H23A	109.0
C6—C4—C5	118.9 (7)	C25—C23—H23A	109.0
C6—C4—P1	118.6 (5)	N2—C23—H23B	109.0
C5—C4—P1	122.5 (6)	C25—C23—H23B	109.0
C15—C13—C12	120.9 (7)	H23A—C23—H23B	107.8
C15—C13—H13	119.6	C18—C19—C11	119.9 (8)
C12—C13—H13	119.6	C18—C19—H19	120.0
C8—C5—C4	119.0 (7)	C11—C19—H19	120.0
C8—C5—H5	120.5	C27—C24—C22	111.0 (7)
C4—C5—H5	120.5	C27—C24—H24A	109.4
C7—C6—C4	120.7 (7)	C22—C24—H24A	109.4
C7—C6—H6	119.7	C27—C24—H24B	109.4
C4—C6—H6	119.7	C22—C24—H24B	109.4
C21—C14—C12	120.5 (7)	H24A—C24—H24B	108.0
C21—C14—H14	119.7	C27—C25—C23	112.4 (7)
C12—C14—H14	119.7	C27—C25—H25A	109.1
C20—C15—C13	121.2 (8)	C23—C25—H25A	109.1
C20—C15—H15	119.4	C27—C25—H25B	109.1
C13—C15—H15	119.4	C23—C25—H25B	109.1
C10—C11—C19	118.6 (8)	H25A—C25—H25B	107.8
C10—C11—H11	120.7	C24—C27—C25	111.1 (7)
C19—C11—H11	120.7	C24—C27—H3A	109.4
C16—C7—C6	120.3 (8)	C25—C27—H3A	109.4
C16—C7—H7	119.8	C24—C27—H3B	109.4
C6—C7—H7	119.8	C25—C27—H3B	109.4
C16—C8—C5	120.8 (7)	H3A—C27—H3B	108.0
			
C17—N1—C10—C11	−0.9 (13)	P1—C4—C6—C7	−178.9 (6)
C17—N1—C10—P1	176.7 (7)	C13—C12—C14—C21	0.4 (10)
C4—P1—C10—N1	−86.6 (6)	P1—C12—C14—C21	176.7 (5)
C12—P1—C10—N1	167.9 (6)	C12—C13—C15—C20	−0.2 (12)
Re1—P1—C10—N1	37.9 (7)	N1—C10—C11—C19	1.1 (13)
C4—P1—C10—C11	90.9 (7)	P1—C10—C11—C19	−176.3 (7)
C12—P1—C10—C11	−14.6 (8)	C4—C6—C7—C16	−0.1 (13)
Re1—P1—C10—C11	−144.5 (6)	C4—C5—C8—C16	1.5 (11)
C4—P1—C12—C14	155.1 (6)	C13—C15—C20—C21	0.3 (12)
C10—P1—C12—C14	−98.9 (6)	C23—N2—C22—C24	58.2 (8)
Re1—P1—C12—C14	32.4 (7)	Re1—N2—C22—C24	−167.6 (5)
C4—P1—C12—C13	−28.7 (6)	C15—C20—C21—C14	0.0 (11)
C10—P1—C12—C13	77.3 (6)	C12—C14—C21—C20	−0.3 (11)
Re1—P1—C12—C13	−151.4 (5)	C5—C8—C16—C7	−1.7 (13)
C12—P1—C4—C6	−58.3 (6)	C6—C7—C16—C8	1.0 (13)
C10—P1—C4—C6	−164.3 (6)	C10—N1—C17—C18	−1.2 (15)
Re1—P1—C4—C6	67.1 (6)	C19—C18—C17—N1	3.1 (15)
C12—P1—C4—C5	123.0 (6)	C22—N2—C23—C25	−56.3 (8)
C10—P1—C4—C5	17.0 (7)	Re1—N2—C23—C25	169.3 (5)
Re1—P1—C4—C5	−111.6 (6)	C17—C18—C19—C11	−2.9 (14)
C14—C12—C13—C15	−0.1 (11)	C10—C11—C19—C18	0.8 (14)
P1—C12—C13—C15	−176.5 (6)	N2—C22—C24—C27	−56.7 (9)
C6—C4—C5—C8	−0.5 (11)	N2—C23—C25—C27	53.0 (10)
P1—C4—C5—C8	178.2 (6)	C22—C24—C27—C25	51.0 (9)
C5—C4—C6—C7	−0.2 (11)	C23—C25—C27—C24	−49.9 (10)

### Bromidotricarbonyl[diphenyl(pyridin-2-yl)phosphane-κP](piperidine-κN)rhenium(I) (II) Hydrogen-bond geometry (Å, º)

**Table:**

*D*—H···*A*	*D*—H	H···*A*	*D*···*A*	*D*—H···*A*
N2—H2*N*···N1	0.82	2.34	2.992 (9)	138
C14—H14···Br1	0.93	2.78	3.586 (8)	146
C22—H22*B*···Br1	0.97	2.83	3.499 (7)	127

## References

[bb1] Abram, U., Alberto, R., Dilworth, J. R., Zheng, Y. & Ortner, K. (1999). *Polyhedron*, **18**, 2995–3003.

[bb2] Bruker (2012). *SMART*, *SAINT* and *SADABS*. Bruker AXS Inc., Madison, Wisconsin, USA.

[bb3] Cannizzo, A., Blanco-Rodríguez, A. M., El Nahhas, A., Šebera, J., Záliš, S., Vlček, A. Jr & Chergui, M. (2008). *J. Am. Chem. Soc.* **130**, 8967–8974. 10.1021/ja710763w 18570416

[bb4] Dalal, J., Sinha, N., Yadav, H. & Kumar, B. (2015). *RSC Adv.* **5**, 57735–57748.

[bb5] Darensbourg, D. J., Andreatta, J. R., Stranahan, S. M. & Reibenspies, J. H. (2007). *Organometallics*, **26**, 6832–6838.

[bb6] Guiry, P. J. & Saunders, C. P. (2004). *Adv. Synth. Catal.* **346**, 497–537.

[bb7] Knebel, W. J. & Angelici, R. J. (1973). *Inorg. Chim. Acta*, **7**, 713–716.

[bb8] Kumar, P., Singh, A. K., Pandey, R. & Pandey, D. S. (2011). *J. Organomet. Chem.* **696**, 3454–3464.

[bb9] Machura, B. & Kruszynski, R. (2006). *Polyhedron*, **25**, 1985–1993.

[bb10] Macrae, C. F., Bruno, I. J., Chisholm, J. A., Edgington, P. R., McCabe, P., Pidcock, E., Rodriguez-Monge, L., Taylor, R., van de Streek, J. & Wood, P. A. (2008). *J. Appl. Cryst.* **41**, 466–470.

[bb11] McKinnon, J. J., Jayatilaka, D. & Spackman, M. A. (2007). *Chem. Commun*. pp. 3814–3816. 10.1039/b704980c 18217656

[bb12] Ooyama, D. & Sato, M. (2004). *Appl. Organomet. Chem.* **18**, 380–381.

[bb13] Pizarro, N., Duque, M., Chamorro, E., Nonell, S., Manzur, J., de la Fuente, J. R., Günther, G., Cepeda-Plaza, M. & Vega, A. (2015). *J. Phys. Chem. A*, **119**, 3929–3935. 10.1021/jp512614w 25853537

[bb14] Sheldrick, G. M. (2008). *Acta Cryst.* A**64**, 112–122. 10.1107/S0108767307043930 18156677

[bb15] Sheldrick, G. M. (2015). *Acta Cryst.* C**71**, 3–8.

[bb16] Spackman, M. A. & Jayatilaka, D. (2009). *CrystEngComm*, **11**, 19–32.

[bb17] Spek, A. L. (2009). *Acta Cryst.* D**65**, 148–155. 10.1107/S090744490804362X PMC2631630 19171970

[bb18] Spek, A. L. (2015). *Acta Cryst.* C**71**, 9–18. 10.1107/S2053229614024929 25567569

[bb19] Turner, M. J., McKinnon, J. J., Wolff, S. K., Grimwood, D. J., Spackman, P. R., Jayatilaka, D. & Spackman, M. A. (2017). *CrystalExplorer17*. University of Western Australia. http://hirshfeldsurface.net

[bb20] Venegas, F., Pizarro, N. & Vega, A. (2011). *J. Chil. Chem. Soc.* **56**, 823–826.

[bb21] Westrip, S. P. (2010). *J. Appl. Cryst.* **43**, 920–925.

